# Core2 *O*-glycan-expressing prostate cancer cells are resistant to NK cell immunity

**DOI:** 10.3892/mmr.2012.1189

**Published:** 2012-11-19

**Authors:** TEPPEI OKAMOTO, MIHOKO SUTOH YONEYAMA, SHINGO HATAKEYAMA, KAZUYUKI MORI, HAYATO YAMAMOTO, TAKUYA KOIE, HISAO SAITOH, KANEMITSU YAMAYA, TOMIHISA FUNYU, MINORU FUKUDA, CHIKARA OHYAMA, SHIGERU TSUBOI

**Affiliations:** 1 Department of Urology, Hirosaki University Graduate School of Medicine, Hirosaki 036-8562; 2 Department of Advanced Transplant and Regenerative Medicine, Hirosaki University Graduate School of Medicine, Hirosaki 036-8562; 3 Department of Biochemistry, Oyokyo Kidney Research Institute, Hirosaki 036-8243, Japan; 4 Department of Urology, Oyokyo Kidney Research Institute, Hirosaki 036-8243, Japan; 5 Department of Glycobiology Unit, Tumor Microenvironment Program, Sanford-Burnham Medical Research Institute, La Jolla, CA 92037, USA

**Keywords:** prostate cancer, metastasis, core2 *O*-glycans, NK cell immunity, MUC1

## Abstract

Core2 β-1,6-*N*-acetylglucosaminyltransferase (C2GnT) forms an *N*-acetylglucosamine branch in the *O*-glycans (core2 *O*-glycans) of cell surface glycoproteins. We previously revealed that the expression of C2GnT is positively correlated with poor prognosis in prostate cancer patients. However, the detailed mechanisms underlying their poor prognosis remain unclear. In the current study, we report that the core2 *O*-glycans carried by the surface MUC1 glycoproteins of prostate cancer cells play an important role in the evasion of NK cell immunity. In C2GnT-expressing prostate cancer cells, the MUC1 core2 *O*-glycans are modified with poly-*N*-acetyllactosamine. MUC1 glycoproteins carrying poly-*N*-acetyllactosamine attenuated the interaction of the cancer cells with NK cells, resulting in decreased secretion of granzyme B by the NK cells. Poly-*N*-acetyllactosamine also interfered with the ability of tumor necrosis factor-related apoptosis-inducing ligand (TRAIL) to access the cancer cell surface. These effects of poly-*N*-acetyllactosamine on NK cells render C2GnT-expressing prostate cancer cells resistant to NK cell cytotoxicity. By contrast, C2GnT-deficient prostate cancer cells carrying a lower amount of poly-*N*-acetyllactosamine than the C2GnT-expressing prostate cancer cells were significantly more susceptible to NK cell cytotoxicity. Our results strongly suggest that C2GnT-expressing prostate cancer cells evade NK cell immunity and survive longer in the host blood circulation, thereby resulting in the promotion of prostate cancer metastasis.

## Introduction

One of the major prognostic determinants of cancer patients is metastasis. The process of metastasis involves multiple steps (1). A growing body of evidence supports crucial roles for cell-surface carbohydrates during the process of metastasis. Cell-surface carbohydrates presented by glycoproteins are classified in accordance with their linkage to proteins and comprise *N*-glycans [*N*-acetylglucosamine (GlcNAc) to asparagine] and *O*-glycans [*N*-acetylgalactosamine (GalNAc) to serine (Ser) or threonine (Thr)]. It has been reported that *N*-glycans are involved in several steps of the metastatic process (2), but the roles of *O*-glycans remain unclear. We have concentrated our efforts on understanding the roles of *O*-glycans in tumor metastasis.

**Figure 1:**
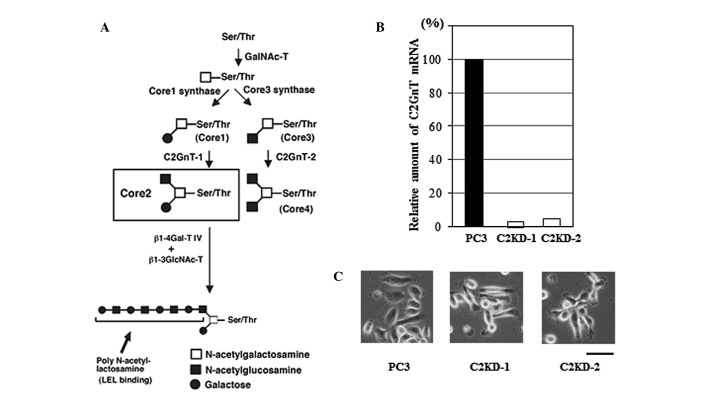
Establishment of core2 β-1,6-*N*-acetylglucosaminyltransferase (C2GnT)-deficient prostate cancer cells. (A) Biosynthesis pathway of *O*-glycan core structures, core1-4. *N*-acetylgalactosamine (GalNAc) is transferred to serine (Ser) or threonine (Thr) residues in a polypeptide by peptide GalNAc transferase (GalNAc-T). GalNAcα1-Ser/Thr is converted by Core1 synthase to Galβ1-3GalNAcα1-Ser/Thr (core1). Core1 is then converted by C2GnT-1 to core2. Core1 is also converted by Core3 synthase to core3. Core3 is converted by C2GnT-2 to core4. β-1,4-galactosyltransferase IV (β1-4Gal-T IV) together with β-1,3-*N*-acetylglucosaminyltransferase (β1-3GlcNAc-T) synthesize poly-*N*-acetyllactosamine in core2 branched oligosaccharides. *Lycoperiscon esculentum* (tomato) lectin (LEL) binds specifically to poly-*N*-acetyllactosamine with at least three lactosamine unit repeats. (B) Relative expression levels of C2GnT in prostate cancer cell lines were analyzed by RT-PCR. (C) Cell morphologies of PC3 and C2GnT-deficient cells. Bar is 50 μm.

Fig. 1A shows the biosynthesis pathway of various types of core structures of *O*-glycans. GalNAc is transferred to Ser and Thr residues in the polypeptide, and then GalNAcα1-Ser/Thr may be extended with various carbohydrates, including galactose (Gal), GlcNAc, fucose or sialic acid. Depending on the carbohydrates added, four common *O*-glycan core structures, core1 through core4, are expressed in mammalian tissues (Fig. 1A). A key enzyme for the formation of *O*-glycans containing an GlcNAc branch linked to GalNAc (GlcNAcβ1-6GalNAc, core2 branch) is designated core2 β-1,6-*N*-acetylglucosaminyltransferase (C2GnT; Fig. 1A). The expression of C2GnT is closely correlated with highly metastatic phenotypes of several types of cancer (3–7).

The core2 branch is a scaffold for the subsequent production of lactosamine disaccharide repeats, specifically poly-*N*-acetyllactosamine (Galβ1-4GlcNAc)n, on *O*-glycans in a wide variety of cells (Fig. 1A) (8). Our previous studies concerning bladder cancer revealed that C2GnT-expressing bladder cancer cell-surface glycoproteins carrying poly-*N*-acetyllactosamine on their *O*-glycans play an important role in bladder cancer metastasis by facilitating the evasion of NK cell immunity by the cancer cells (7,9,10).

Prostate cancer is the most common cancer in males and the second leading cause of male cancer mortality in the US and Western world, mainly due to metastatic disease (11). Our previous histological analyses of patient specimens revealed that the expression of C2GnT is positively correlated with poor prognosis in prostate cancer patients (6). We hypothesized that C2GnT-overxpressing prostate cancer cells acquire the highly metastatic phenotype by evading NK cell immunity. In this study, we examined how C2GnT-expressing prostate cancer cells evade NK cell immunity.

## Materials and methods

### Cells, reagents and antibodies

PC3, a highly metastatic prostate cancer cell line, was obtained from American Type Culture Collection (ATCC; Manassas, VA, USA). Cells were maintained in RPMI-1640 medium (Sigma-Aldrich, St. Louis, MO, USA) supplemented with 10% fetal bovine serum (FBS) (PAA Laboratories, Morningside, Australia). All biochemical reagents were purchased from Sigma-Aldrich, unless otherwise noted. Recombinant human tumor necrosis factor-related apoptosis-inducing ligand (TRAIL) was purchased from R&D Systems, Inc. (Minneapolis, MN, USA). The following monoclonal antibodies to human antigens were used: anti-MUC1 (VU4H5; Cell Signaling Technology, Danvers, MA, USA) and phycoerythrin (PE)-labeled anti-CD56 (BD Biosciences, San Jose, CA, USA). The following polyclonal antibodies were used: anti-lysosome-associated membrane glycoprotein 1 (anti-LAMP1; Lifespan Biosciences, Seattle, WA, USA), anti-death receptor 4 (anti-DR4; Millipore, Temecula, CA, USA) and anti-actin (Sigma-Aldrich).

### Stable transfectants

PC3 cells express high levels of C2GnT. PC3 cells with reduced expression levels of C2GnT were generated using shRNA technology as previously described (7). An shRNA expression plasmid was constructed using pBAsi-hU6 Neo DNA (Takara Bio Inc., Shiga, Japan). The shRNA sequence for C2GnT-1 was: GATCCGAATCCTAGTAGT GATATTTAGTGCTCCTGGTTGAATATCACTACTAGG ATTCTTTTTTA. The siRNA sequence is underlined. The sequence of the control shRNA containing the scrambled siRNA sequence was: GATCCGTCTTAATCGCGTATAAGG CTAGTGCTCCTGGTTGGCCTTATACGCGATTAAGAC TTTTTTA. The shRNA expression plasmids (knockdown and control constructs) were introduced into PC3 cells using Lipofectamine 2000. Drug-resistant colonies were selected in the presence of 200 μg/ml geneticin. Total RNA was prepared from each drug-resistant cell line using a FastPure RNA kit (Takara Bio Inc.). Quantitative real-time PCR was performed to quantify the C2GnT mRNA level of each drug-resistant cell line using a Prime Script RT-PCR kit and Thermal Cycler Dice Real Time system (Takara Bio Inc.). The sequences of the primer set used for C2GnT-1 were: 5′-GGATGTCACCTG GAATCAGCACTA-3′ and 5′-TTATCAGAGCTGCAACGG CATC-3′. The primers specific for glyceraldehyde-3-phosphate dehydrogenase (GAPDH) were: 5′-ATGACTCTACCCACG GCAAG-3′ and 5′-CATACTCAGCACCAGCATCAC-3′ and were used as the internal control. Two C2GnT-deficient clones (C2KD-1 and -2) were chosen based on their reduced mRNA expression levels. C2KD-1, C2KD-2 and one control clone (PC3) were used for the assays described in the present study.

### Western blotting

Total lysates of cancer cells were prepared by solubilization in 50 mM Tris-HCl buffer, pH 7.5, containing 1% IgepalCA-630, 150 mM NaCl and proteinase inhibitors. The lysates were resolved by SDS-PAGE on an 8–16% gradient gel (Invitrogen Life Technologies, Carlsbad, CA, USA) and transferred to a polyvinylidene fluoride (PVDF) membrane. Western blotting analyses were performed using specific primary antibodies and a horseradish peroxidase-conjugated secondary antibody. Signals were visualized using the ECL PLUS detection system (GE Healthcare, Amersham, UK).

### Immunoprecipitation

Lectin immunopreocipitation was performed as previously described (7). For lectin immunoprecipitation, lysates from prostate cancer cells were incubated with *Lycoperiscon esculentum* (tomato) lectin (LEL)-agarose (Vector Laboratories, Burlingame, CA, USA). The resin binding the immune complex was eluted with 1X Laemmli SDS-PAGE sample buffer.

### Preparation of NK cells

Human primary NK cells were purified from human peripheral blood mononuclear cells using an NK cell isolation kit (Myltenyi Biotech, Auburn, CA, USA). The NK cells (1×10^6^ cells/ml) were cultured for 3 days in RPMI-1640 medium supplemented with 10% FBS in the presence of 1000 U/ml human recombinant IL-2 (Wako, Osaka, Japan).

### Conjugate formation and granzyme B secretion assay

Heterotypic cell conjugates were quantitatively determined by a double fluorescence assay (12) with certain modifications. Prostate cancer target cells (2×10^6^ cells/ml) were tranfected with a green fluorescence protein (GFP)-expression plasmid, pmaxGFP (Lonza Walkersville, Inc., Walkersville, MD, USA). After 1 h incubation of the GFP-expressing tumor cells with IL-2-activated NK cells at 37°C, the cells were stained with PE-labeled anti-CD56 antibody and then the number of conjugates were counted using a flow cytometer, FACScant II (BD Biosciences). Double-colored (green and red) conjugates were calculated as a percentage of the total GFP-positive cells. For the granzyme B secretion assay, the culture supernatant was collected from the co-culture of the target cancer cells with NK cells. The quantity of granzyme B in the supernatant was assayed by measuring serine proteinase activity with a colorimetric peptide substrate, IEPD-*p*-nitroanilide (Biomol International, Plymouth Meeting, PA, USA) as previously described (7). Specific granzyme B secretion was expressed as a percentage of the total cellular enzyme activity after subtracting the spontaneous release.

### NK cytotoxicity assay

Cytotoxicity was measured using the Cytotox 96 Non-radioactive Cytotoxicity Assay kit (Promega Corporation, Madison, MI, USA). The target cells (tumor cells) were incubated with IL-2-activated NK cells for 4 h at 37°C. The release of lactate dehydrogenase from the lysed target cells was measured. All assays were performed in quadruplicate. Percentage cytotoxicity = (experimental lactate dehydrogenase release - effector spontaneous release - target spontaneous release)/(target maximum release - target spontaneous release) × 100.

### Cell viability assay

For the cell viability assay, the cells (1×10^4^ cells/well in 96-well plates) were incubated with the indicated concentration of recombinant TRAIL at 37°C for the indicated time. The cell viability was assayed using the Cell Counting Kit-8 (Dojindo Laboratories, Kumamoto, Japan) according to the manufacturer’s instructions.

### Statistical analysis

We used the statistical program SPSS 12.0 (SPSS, Chicago, IL, USA). Statistically significant differences were determined using the Student’s t-test. P<0.05 was considered to indicate a statistically significant result.

## Results

### Establishment of C2GnT-deficient prostate cancer cells

C2GnT is responsible for the formation of core2 *O*-glycans (Fig. 1A). To test our hypothesis, we investigated the role of core2 *O*-glycans in the evasion of NK cell immunity by C2GnT-expressing prostate cancer. We used a prostate cancer cell line, PC3, which is derived from a malignant and metastatic prostate cancer and expresses C2GnT at a high level (13). We established two C2GnT-deficient cell lines (designated C2KD-1 and C2KD-2) and one control cell line (PC3) using PC3 cells. RT-PCR analysis of the C2GnT expression showed that the C2GnT expression levels were markedly reduced in the C2KD-1 and C2KD-2 cells compared with those in the PC3 cells (Fig. 1B). These cells exhibited no marked morphological differences (Fig. 1C). The results obtained for C2KD-1 and PC3 only are shown, since the two C2GnT-deficient clones yielded almost identical results in all assays.

### Core2 O-glycosylation of MUC1

**Figure 2:**
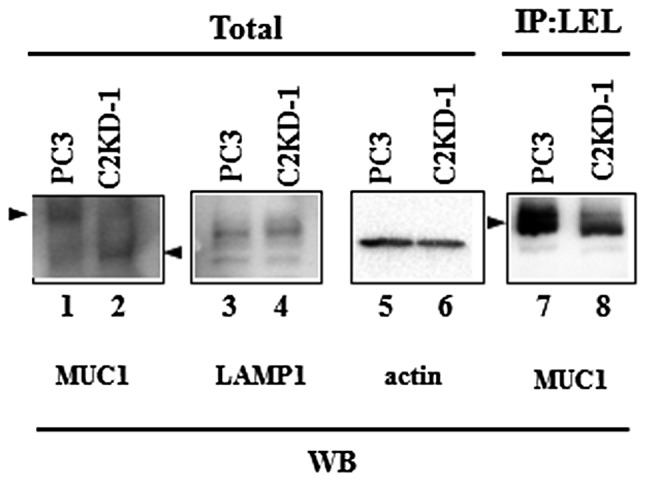
Core2 *O*-glycosylation of MUC1 in core2 β-1,6-*N*-acetylglucosaminyltransferase (C2GnT)-expressing prostate cancer cells. Total cell lysates from PC3 and C2GnT-deficient PC3 (C2KD-1) prostate cancer cells, were analyzed by western blotting with anti-MUC1 (lanes 1 and 2), anti-lysosome-associated membrane glycoprotein 1 (anti-LAMP1) (lanes 3 and 4) and anti-actin (lanes 5 and 6) as a sample loading control. Total lysates were immunoprecipitated with *Lycoperiscon esculentum* lectin (LEL)-agarose followed by western blotting analysis with anti-MUC1 (lanes 7 and 8).

We have previously demonstrated that patients with C2GnT-expressing prostate cancer have a significantly shorter survival time than patients with C2GnT-non-expressing prostate cancer (6), suggesting that C2GnT-expressing prostate cancer is highly metastatic. However, a growing body of evidence indicates that *O*-glycans in cell-surface mucins are important in numerous biological processes, including the protection of epithelial cell surfaces, immune response, cell adhesion, inflammation, tumorigenesis and tumor metastasis (14). These observations led us to postulate that core2 *O*-glycans carried by mucins play an important role in prostate cancer metastasis. We analyzed the MUC1 glycoproteins of the prostate cancer cells for *O*-glycosylation by western blotting. The MUC1 of the control PC3 cells exhibited a higher molecular weight than the MUC1 from C2GnT-deficient cells (C2KD-1; Fig. 2, lanes 1 and 2). By contrast, we observed no significant differences in the molecular weights of the non-*O*-glycosylated cell-surface protein, LAMP1, between the PC3 and C2KD-1 cells (Fig. 2, lanes 3 and 4). These results indicate that MUC1 glycoproteins from PC3 cells carry higher levels of core2 *O*-glycans than those from C2KD-1 cells due to their higher expression levels of C2GnT.

The core2 branch is a scaffold for the subsequent production of lactosamine disaccharide repeats, specifically poly-*N*-acetyllactosamine (Galβ1-4GlcNAc)n (Fig. 1A) (8). To determine whether MUC1 from PC3 cells carries poly-*N*-acetyllactosamine on its *O*-glycans, we first excluded *N*-glycans from prostate cancer cells by treatment with tunicamycin, an *N*-glycosylation inhibitor, and then analyzed the cell lysates by immunoprecipitation using LEL. LEL binds specifically to poly-*N*-acetyllactosamines with at least three lactosamine unit repeats. The LEL immunoprecipitates were subjected to western blotting with anti-MUC1 antibody. MUC1 was detected in the LEL immunoprecipitates from the PC3 cells, but the amount of MUC1 detected in the LEL immunoprecipitates from the C2KD-1 cells was markedly reduced (Fig. 2, lanes 7 and 8). This result indicates that MUC1 from C2GnT-expressing PC3 cells carries a larger amount of poly-*N*-acetyllactosamine on its core2 *O*-glycans than that from C2KD-1 cells.

### Effects of poly-N-acetyllactosamine on NK cell functions

**Figure 3:**
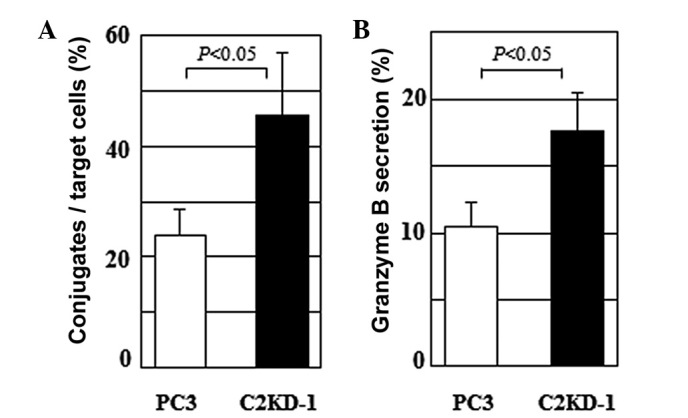
Effects of poly-*N*-acetyllactosamine on NK cell functions (A) Conjugate formation of NK cells with PC3 (open bar) and C2GnT-deficient PC3 (C2KD-1; closed bar) cells. (B) Granzyme B secretion by NK cells upon stimulation with the NK cell-target prostate cancer cell interaction was measured by assaying the protease activity of granzyme B in the co-culture supernatants of PC3 (open bar) and C2KD-1 (closed bar) cells. Specific granzyme B secretion was expressed as a percentage of the total cellular enzyme activity after subtracting the spontaneous release. Mean values ± SE of three independent experiments.

NK cells play a critical role in tumor rejection responses in the host blood circulation. The attack on cancer cells by NK cells is initiated by the NK cell-cancer cell interaction mediated through the NK receptor-ligand interaction (15). To determine whether C2GnT expression affects the NK cell-prostate cancer cell interaction, we compared the conjugate formation of NK cells with PC3 and C2KD-1 cells. Following the incubation of the target cells with NK cells for 1 h, the NK cells formed a significantly higher number of conjugates with the C2KD-1 cells than with the PC3 cells (Fig. 3A). This result, taken together with Fig. 2, suggests that in the C2GnT-expressing cells (PC3), the poly-*N*-acetyllactosamine moieties carried by the MUC1 core2 *O*-glycans reduce the adhesive properties of MUC1 due to their bulkiness, resulting in reduced conjugate formation between the PC3 and NK cells (Fig. 3A).

NK cells are activated through the NK cell-target cell interaction and release their granular contents to kill the target cells. The granular contents include perforin and granzyme B which induce apoptosis of the target cells. We measured the secretion of granzyme B stimulated by the NK cell-prostate cancer cell interaction using an assay of the protease activity of granzyme B in the co-culture supernatant. The C2GnT-expressing PC3 cells induced the secretion of significantly lower levels of granzyme B than the C2GnT-deficient C2KD-1 cells (Fig. 3B). These results suggest that MUC1 glycoproteins carrying poly-*N*-acetyllactosamine attenuate the NK cell-prostate cancer cell interaction, resulting in decreased secretion of granzyme B (Fig. 3).

### Effect of poly-N-acetyllactosamine on NK cell cytotoxicity

**Figure 4:**
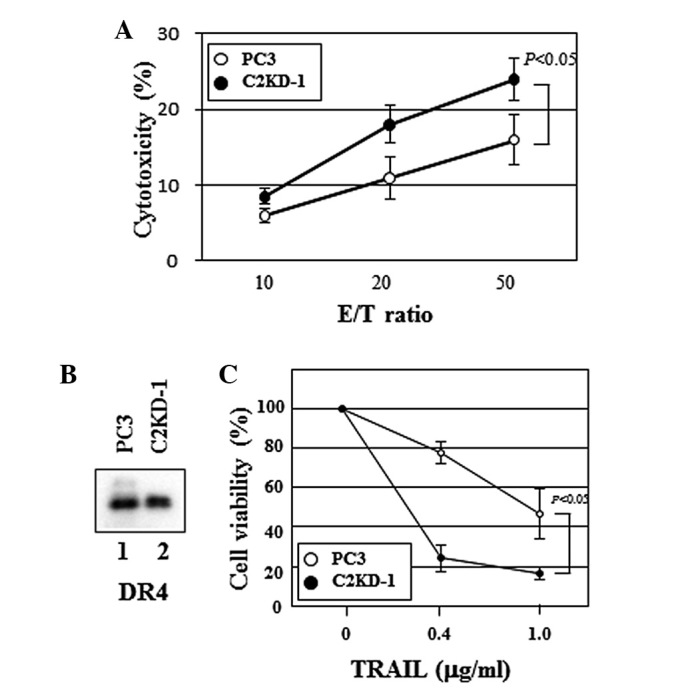
Effect of core2 β-1,6-*N*-acetylglucosaminyltransferase (C2GnT) expression on NK cell cytotoxicity. (A) Cytotoxicity of human NK cells against PC3 (open circles) and C2GnT-deficient PC3 (C2KD-1; closed circles) was assayed. (B) Expression of death receptor 4 (DR4), a receptor for tumor necrosis factor-related apoptosis-inducing ligand (TRAIL) in the PC3 (lane 1) and C2KD-1 (lane 2) cells was analyzed by western blotting. (C) Cell viability of PC3 (open circles) and C2KD-1 (closed circles) cells. Cells were incubated with soluble recombinant TRAIL for 24 h at the indicated concentrations. Cell viability was determined using Cell Counting Kit-8. Mean values ± SE of three independent experiments.

We then questioned whether C2GnT expression by prostate cancer cells affects the cytotoxic activity of NK cells, since the interaction of NK cells with C2GnT-expressing prostate cancer cells was impaired and the secretion of the target cell apoptosis-inducing substance, granzyme B was reduced (Fig. 3). To address this, we assayed the cytotoxicity of NK cells against prostate cancer cells. Fig. 4A shows that NK cells killed C2KD-1 cells more efficiently than PC3 cells (Fig. 4A), indicating that C2GnT-expressing prostate cancer cells (PC3) were more resistant to NK cell cytotoxicity than were C2GnT-deficient cells (C2KD-1).

NK cells use two main mechanisms to kill tumor cells. One is that NK cells are activated through the NK cell receptor-tumor ligand interaction to release cytotoxic granules, including perforin and granzymes. The other is that NK cells kill tumor cells using death ligands, including TRAIL (16). TRAIL induces target cell apoptosis through its interaction with death receptors, including DR4, on the surface of the target cell. We evaluated the expression of DR4 on the prostate cancer cells by western blotting. We observed no significant differences in the DR4 expression levels between PC3 and C2KD-1 (Fig. 4B, lanes 1 and 2). Unlike MUC1, no significant differences in the *O*-glycosylation levels of DR4 between PC3 and C2KD-1 cells were observed on western blots (Figs. 2 and 4B). To evaluate the effect of C2GnT expression on the sensitivity of prostate cancer cells to TRAIL, we measured TRAIL-induced cell death using soluble recombinant TRAIL. In the presence of TRAIL, the viability of the PC3 cells was significantly higher than that of the C2KD-1 cells (Fig. 4C), indicating that C2GnT-expressing prostate cancer cells are more resistant to TRAIL than are C2GnT-deficient cells.

## Discussion

**Figure 5:**
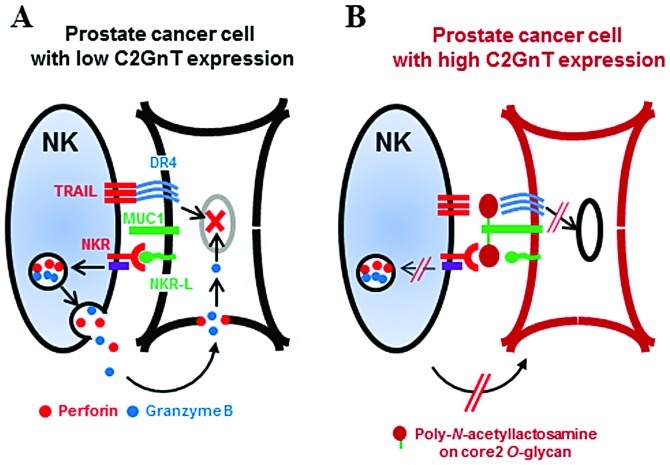
Schematic drawing of the evasion of NK cell immunity by core2 β-1,6-*N*-acetylglucosaminyltransferase (C2GnT)-expressing prostate cancer cells. (A) Prostate cancer cells with low or no C2GnT expression are susceptible to NK cell immunity. NK cells are activated by the interaction of the NK receptor (NKR) with its ligand (NKR-L) expressed on the surface of cancer cells. Activated NK cells secrete perforin and granzyme B to induce the apoptosis of target cancer cells. In addition, tumor necrosis factor-related apoptosis-inducing ligand (TRAIL) expressed in NK cells interacts with death receptors expressed in tumor cells such as death receptor 4 (DR4) to induce the apoptosis of target tumor cells. (B) Prostate cancer cells with high C2GnT expression levels are resistant to NK cell immunity. Poly-*N*-acetyllactosamine carried by MUC1 core2 *O*-glycans attenuates the NK cell-cancer cell interaction due to its bulkiness. The attenuated interaction results in decreased NK cell degranulation and also interferes with the access of TRAIL to DR4, impairing the TRAIL-mediated killing of target tumor cells. This shielding of MUC1 carrying core2 *O*-glycans and poly-*N*-acetyllactosamine increases the survival of the C2GnT-expressing prostate cancer cells in the host blood circulation, promoting the prostate cancer metastasis.

We have previously shown that MUC1 is modified by poly-*N*-acetyllactosamine on its *O*-glycan residues in C2GnT-expressing prostate cancer cells. MUC1 is significant in adhesion as it is one of the molecules that extends most highly above the cell surface (14). Our results taken together with this observation suggest that the modification of MUC1 in C2GnT-expressing prostate cancer cells with bulky poly-*N*-acetyllactosamine moieties reduces their adhesiveness, thereby attenuating the NK cell-cancer cell interaction. The attenuated interaction results in decreased degranulation by NK cells and reduction of the accessibility of TRAIL to the death receptors (Fig. 5). These effects allow C2GnT-expressing prostate cancer cells to evade NK cell immunity in the circulation. In our previous study, when bladder tumor cells were intravenously injected into nude mice, C2GnT-expressing tumor cells produced a greater number of metastatic foci in the lungs than were produced by C2GnT-non-expressing tumor cells (7). Our present data taken together with the previous observations strongly suggest that this immune evasion results in longer survival of C2GnT-expressing prostate cancer cells in the host blood circulation, resulting in the promotion of prostate cancer metastasis.

Wagner *et al* reported that tumor-cell sensitivity to TRAIL was controlled by *O*-glycosylation of death receptors (DR4 and DR5). In 22 out of 28 TRAIL-sensitive cancer cell lines, the expression levels of a peptidyl *O*-glycosyltransferase which catalyzes the initial step of *O*-glycosylation were elevated (17). It was shown that *O*-glycosylation of death receptors promoted TRAIL-stimulated clustering of the receptors, mediating recruitment and activation of the apoptosis-initiating protease, caspase-8. Although their *O*-glycan structures were not analyzed, *O*-glycosylation increased the TRAIL-sensitivity of the cancer cells. The authors observed *O*-glycosylation of death receptors on the western blots of 22 of the 28 TRAIL-sensitive cell lines. However, no significant *O*-glycosylation of DR4 was observed in the present study, suggesting that C2GnT-expressing prostate cancer cells use a different mechanism to control cancer cell-sensitivity to TRAIL from that reported by Wagner *et al*.

Our investigation provides a new insight into the roles of the carbohydrates carried by tumor cell-surface mucins in tumor metastasis. Tumor cells take advantage of the functions of mucins to maintain homeostasis and promote their survival in variable conditions. Further analyses of the roles of tumor cell-surface carbohydrates are likely to contribute to a better understanding of the tumor metastasis process.
